# Prediction and risk assessment of sepsis-associated encephalopathy in ICU based on interpretable machine learning

**DOI:** 10.1038/s41598-022-27134-6

**Published:** 2022-12-31

**Authors:** Xiao Lu, Hongyu Kang, Dawei Zhou, Qin Li

**Affiliations:** 1 Department of Biomedical Engineering, School of Life Science, Beijing Institute of Technology, Beijing, 100081 China; 2 Department of Critical Care Medicine, Beijing Tongren Hospital, Capital Medical University, Beijing, 100005 China; 3 Institute of Medical Information, Chinese Academy of Medical Sciences and Peking Union Medical College, Beijing, 100020 China

**Keywords:** Medical research, Risk factors, Mathematics and computing

## Abstract

Sepsis-associated encephalopathy (SAE) is a major complication of sepsis and is associated with high mortality and poor long-term prognosis. The purpose of this study is to develop interpretable machine learning models to predict the occurrence of SAE after ICU admission and implement the individual prediction and analysis. Patients with sepsis admitted to ICU were included. SAE was diagnosed as glasgow coma score (GCS) less than 15. Statistical analysis at baseline was performed between SAE and non-SAE. Six machine learning classifiers were employed to predict the occurrence of SAE, and the adjustment of model super parameters was performed by using Bayesian optimization method. Finally, the optimal algorithm was selected according to the prediction efficiency. In addition, professional physicians were invited to evaluate our model prediction results for further quantitative assessment of the model interpretability. The preliminary analysis of variance showed significant differences in the incidence of SAE among patients with pathogen infection. There were significant differences in physical indicators like respiratory rate, temperature, SpO_2_ and mean arterial pressure (*P* < 0.001). In addition, the laboratory results were also significantly different. The optimal classification model (XGBoost) indicated that the best risk factors (cut-off points) were creatinine (1.1 mg/dl), mean respiratory rate (18), pH (7.38), age (72), chlorine (101 mmol/L), sodium (138.5 k/ul), SAPSII score (23), platelet count (160), and phosphorus (2.4 and 5.0 mg/dL). The ranked features derived from the best model (AUC is 0.8837) were mechanical ventilation, duration of mechanical ventilation, phosphorus, SOFA score, and vasopressin usage. The SAE risk prediction model based on XGBoost created here can make very accurate predictions using simple indicators and support the visual explanation. The interpretable model was effectively evaluated by professional physicians and can help them predict the occurrence of SAE more intuitively.

## Introduction

Sepsis is the main cause of ICU morbidity and mortality worldwide, defined as organic dysfunction caused by the hosts’ uncontrolled inflammatory response to an infection^1^. There are multiple complications in sepsis, among which Sepsis-Associated Encephalopathy (SAE) is one of the important clinical manifestations, about 30%-70% of sepsis patients may develop SAE^2^. SAE is a diffuse cerebral dysfunction with a combination of neuroinflammation, vascular changes, and metabolic failure pathophysiology, which is associated with increased short-term mortality, prolonged hospitalization time, or overmuch assumption of medical resources^3,4^. And the diagnosis and treatment impose a heavy medical and economic burden on families and society.

Although the exact mechanism of cerebral dysfunction is still not well understood, it is known that the symptoms of SAE can vary from delirium to coma, and long-term impairment of behavior, memory, and cognitive function may still exist one year after the patient's discharge^5–7^. Due to the lack of standardized criteria, the current clinical diagnosis of SAE usually combines the epidemiological presentation with a variety of ancillary tests, including neurological examination and delirium assessment, neuroimaging, electroencephalography, and biomarkers^8^. However, the early diagnosis rates are still low. Therefore, it is of great significance to identify SAE patients which can not only facilitate timely medical intervention but also improve treatment and prognosis.

With the development of big data analysis, new methodological approaches to identify sepsis and its complications are available. Many hospitals have already established electronic medical record (EHR) based on sepsis monitoring and alert systems to improve early detection and intervention^9^. With machine learning, studies can be divided into SAE prediction and risk factors analysis^10–13^. Sonneville et al.^12^ analyzed potentially modifiable risk factors for SAE at ICU admission based on multivariate logistic regression and developed a Cox proportional hazard model to investigate its impact on 30-day mortality. The results demonstrated that acute renal failure and metabolic disturbances are potentially modifiable factors contributing to SAE and are of great value for the treatment and prognosis of SAE. Zhao et al.^13^, on the other hand, further evaluated the impact of oxygenation status on the patient with SAE and clarified that the optimal range of SpO_2_ for SAE patients is 93%-96%. In the literature of Yang et al.^14^, a nomogram in predicting the 30-day mortality of patients with SAE was obtained by the training set, then internal validation and sensitivity analyses were conducted. And it was revealed that the predictive nomogram had better discrimination than Sequential Organ Failure Assessment (SOFA) and Logistic Organ Dysfunction System (LODS). Nevertheless, high-quality data were required in Yang’s study, and the robustness of the model needed further validation. Although there are a large number of machine learning models to predict sepsis in the retrospective cohorts, model prediction results need to be interpreted and the performance of the models is expected to improve^15^. Even though machine learning models could significantly improve accuracy, they are essentially equivalent to a black box in the prediction process. The final output is an end-to-end prediction that does not allow some non-specialists to understand the decision process clearly ^16,17^.

In recent years, machine learning has been widely used in medical research, especially in the prediction of diseases in ICU^18,19^. In our study, multidimensional feature data were employed to build several machine learning classifiers for predicting the occurrence of SAE within 24 h of ICU admission, and ultimately to select the optimal one. In addition, we conducted an interpretability analysis of the machine learning models which solved the problem that model predictions were “black box”, and enabled the creation of interpretable visualizations of the input and output of a single patient while improving model efficiency, and can be handed over to specialized ICU physicians for quantitative evaluation.

## Methods

### Data source and study population

The retrospective cohort study was conducted from the Medical Information Mart for Intensive Care (MIMIC-IV) open source clinical database, which consisted of more than 40,000 patients in ICU between 2008 and 2019 at Beth Israel Deaconess Medical Center^20^.The MIMIC-IV database can be freely utilized after successful application and ethical approval from the Institutional Review Boards of both Beth Israel Deaconess Medical Center (Boston, MA, USA) and the Massachusetts Institute of Technology (Cambridge, MA, USA).

**Figure 1:**
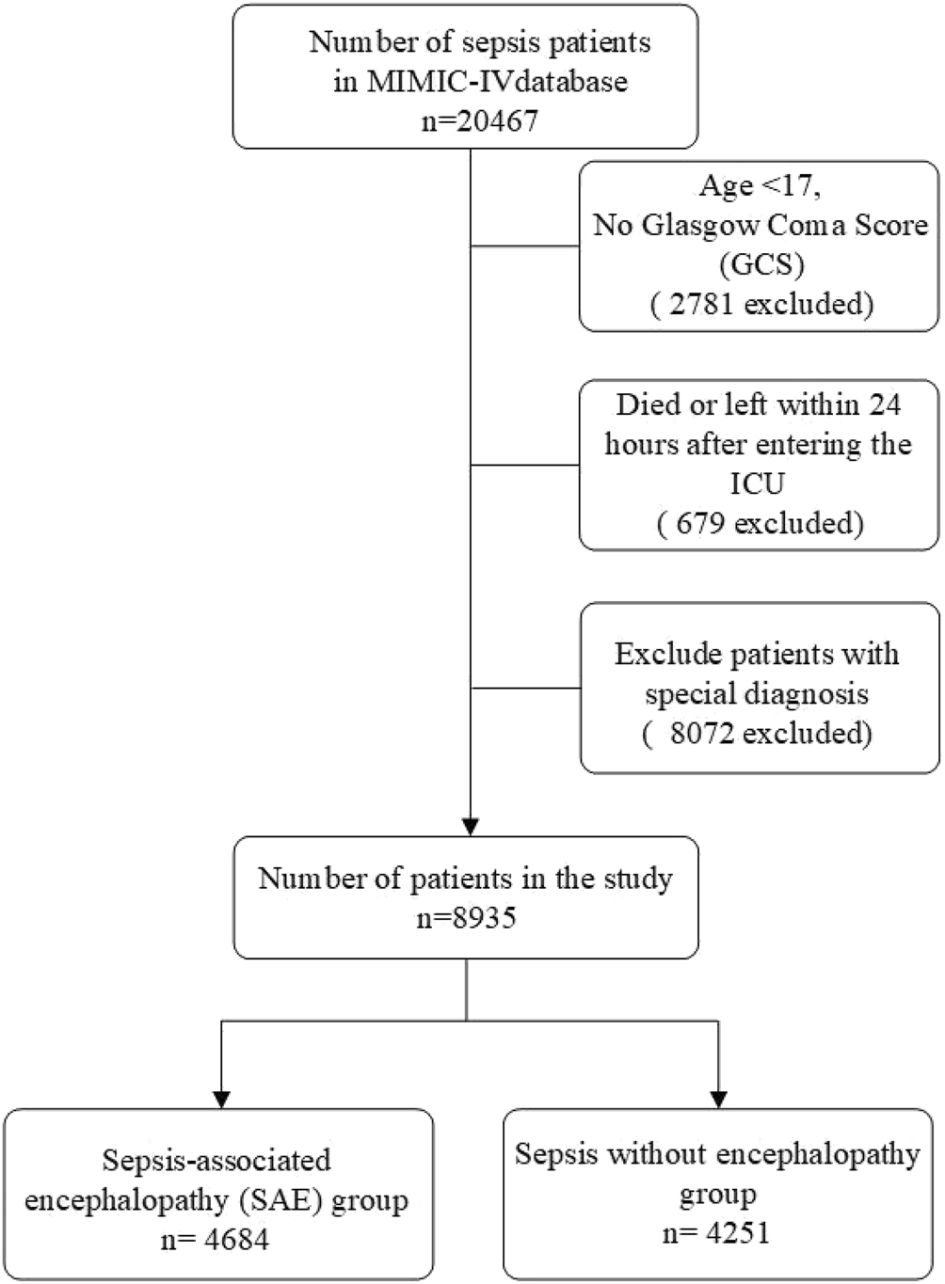
Flow chart of the study population enrollment. The special diagnostics include primary brain injury (traumatic brain injury, ischemic stroke, hemorrhagic stroke, epilepsy, or intracranial infection), severe burn and trauma, chronic alcohol or drug abuse, severe electrolyte imbalances, hyponatremia, hyperglycemia, hypoglycemia, with pre-existing liver or kidney failure affecting consciousness, receiving cardiac resuscitation recently.

SAE is defined as the sepsis patients who have a Glasgow Coma Scale (GCS) ≤ 14 or delirium (according to the ICD-9 code (2930, 2931)). The delirium caused by alcohol or drug abuse, dimension, mental disorders, and neurological diseases were excluded. GCS was considered an important determinant for characterizing SAE and distinguishing it from sepsis^14^.Our study included patients based on the Third International Consensus Definitions for Sepsis (Sepsis-3): (i) Patients with infection confirmed by the positive results of microbial cultivation and (ii) the Sequential Organ Failure Assessment (SOFA) score ≥ 2^21^. Excluded were patients^14^: (i) with primary brain injury (traumatic brain injury, ischemic stroke, hemorrhagic stroke, epilepsy, or intracranial infection); (ii) with pre-existing liver or kidney failure affecting consciousness; (iii) with severe burn and trauma; (iv) receiving cardiac resuscitation recently; (v) with chronic alcohol or drug abuse; (vi) with severe electrolyte imbalances or blood glucose disturbances, including hyponatremia (< 120 mmol/l), hyperglycemia (> 180 mg/dl), or hypoglycemia (< 54 mg/dl); (vii) dying or leaving within 24 h since ICU admission; (viii) without an evaluation of GCS; (ix) < 17 years of age. Eligible patients were enrolled into the final cohort for investigation, and the specific data inclusion analysis process was illustrated in Fig. 1.

56 features were extracted from all patients, including categorical variables such as comorbidities, mechanical ventilation, and the first care unit category within 24 h of admission to the ICU, along with continuous variables such as laboratory tests, vital signs, and demographic characteristics. The completeness of the features we chose was above 80%, and we used multiple interpolation^22^ methods to fill in the missing value. The categorical variables were specially processed in advance, and the numerical transformation was performed to 0,1 categories. All classification variables include gender, ethnicity, first care unit, comorbidity, microorganizations, mechanical utilization, and vaporizer. As shown in the statistical list of Table 1, we used 0,1 to represent the variables that cannot be represented by specific values. For example, we will mark the patients with hypertension as 1 in advance, and the patients without hypertension as 0, so that the classification variables can be handled in advance before entering the model. All variables were normalized (0–1 range) before entering the model. For some indicators which had more than one measurement a day, we calculated the mean, maximum and minimum values to reflect the information of patients in more detail.

**Table 1:** Patients’ baseline clinical characteristics at ICU admission.

Characteristics	Non-SAE group (n = 4251)	SAE group (n = 4684)	*p* value
**Demographic**			
Gender (Male), n (%)	1107 (26.0%)	1206 (25.7%)	0.249
Age, years	66.33 ± 16.26	68.10 ± 16.44	< 0.001
**Ethnicity n (%)**			0.007
White	3073 (72.3%)	3269 (69.8%)	
Black	434 (10.2%)	417 (8.9%)	
Asian	89 (2.1%)	112 (2.4%)	
Hispanic or Latino	89 (2.1%)	94 (2.0%)	
Others	565 (13.3%)	796 (17.0%)	
**First care unit, n (%)**			< 0.001
MICU	1908 (44.9%)	1881 (40.2%)	
TSICU	357 (8.4%)	634 (13.5%)	
CSRU	721 (17.0%)	682 (14.5%)	
CCU	615 (14.5%)	585 (12.5%)	
SICU	646 (15.2%)	905 (19.3%)	
**Severe score**			
SAPSII	21.88 ± 9.54	29.04 ± 11.20	< 0.001
GCS	15.00 ± 0.00	10.42 ± 2.74	< 0.001
SOFA	4.27 ± 2.15	5.58 ± 2.71	< 0.001
**Comorbidity, n (%)**			
Hypertension	1597 (37.6%)	1734 (37.0%)	0.69
Diabetes	1306 (30.7%)	1233 (26.3%)	0.001
Hypothyroidism	431 (10.1%)	472 (10.1%)	0.936
Coagulopathy	163 (3.8%)	203 (4.3%)	0.392
**Physical**			
Mean Heartrate (min^−1^)	88.07 ± 15.26	88.63 ± 16.15	0.201
RR (min^−1^)	20.00 ± 4.25	18.91 ± 4.46	< 0.001
Temperature (°C)	36.89 ± 0.61	37.10 ± 0.71	< 0.001
SpO2 (%)	97.08 ± 2.06	97.54 ± 3.35	< 0.001
Mean arterial pressure (mmHg)	76.97 ± 11.31	77.78 ± 10.74	0.001
**Laboratory results**			
Creatinine(mg/dl)	1.62(0.7–1.5) *	1.40 (0.7–1.3) *	< 0.001
Phosphorus (mg/dl)	3.57 ± 1.27	3.57 ± 1.31	0.938
Chlorine (mmol/L)	104.60 ± 5.99	106.04 ± 6.15	< 0.001
Sodium (K/uL)	138.28 ± 4.14	139.44 ± 4.93	< 0.001
Potassium (K/uL)	4.15 ± 0.71	4.06 ± 0.64	< 0.001
Glucose (mg/dl)	141.87 ± 59.89	147.97 ± 60.86	< 0.001
Platelet Count	231.56 (144.0–288.5) *	239.83(148.25–304.0) *	0.03
White blood cell count(K/uL)	12.92 (8.0–15.6) *	13.89 (8.8–17.1) *	< 0.001
Red blood cell	3.51 ± 0.86	3.54 ± 0.65	0.02
PCO2 (mmHg)	42.68 ± 10.62	42.79 ± 12.67	0.18
pH	7.38 ± 0.07	7.37 ± 0.09	0.274
BUN(K/uL)	25.38 ± 17.35	25.61 ± 17.44	0.751
Hemogloblin(g/dL)	10.42 ± 1.73	10.59 ± 2.11	0.009
PT(s)	15.61 ± 5.53	15.45 ± 4.87	0.884
RDW (%)	15.57 ± 2.29	15.33 ± 2.20	< 0.001
MCV (fL)	89.28 ± 6.92	89.95 ± 6.52	< 0.001
**Microorganisms, n (%)**			
Gram-positive	623 (23.6%)	747 (35.8%)	< 0.001
Gram-negative	754 (28.5%)	890 (42.7%)	< 0.001
Fungus	135 (5.1%)	166 (8.0%)	< 0.001
**Others, n (%)**			
Mechanical ventilation n (%)	740 (28.0%)	1078 (51.7%)	< 0.001
Vasopressor n (%)	805 (30.5%)	827 (39.7%)	< 0.001
ICU stay time, days n (%)	4.68(1.78–4.70) *	9.60 (3.08–12.55) *	< 0.001

*Presented as median (interquartile range).

CCU coronary care unit, CSRU cardiac surgical intensive care unit, MICU medical intensive care unit, SICU surgical intensive care unit, TSICU trauma/surgical intensive care unit, SOFA Sequential Organ Failure Assessment, SAPSII the simplified acute physiology score, GCS Glasgow Coma Score, RDW red blood cell distribution widths, MCV mean corpuscular volume, RR respiratory rate.

### Classification model and model interpretation

**Figure 2:**
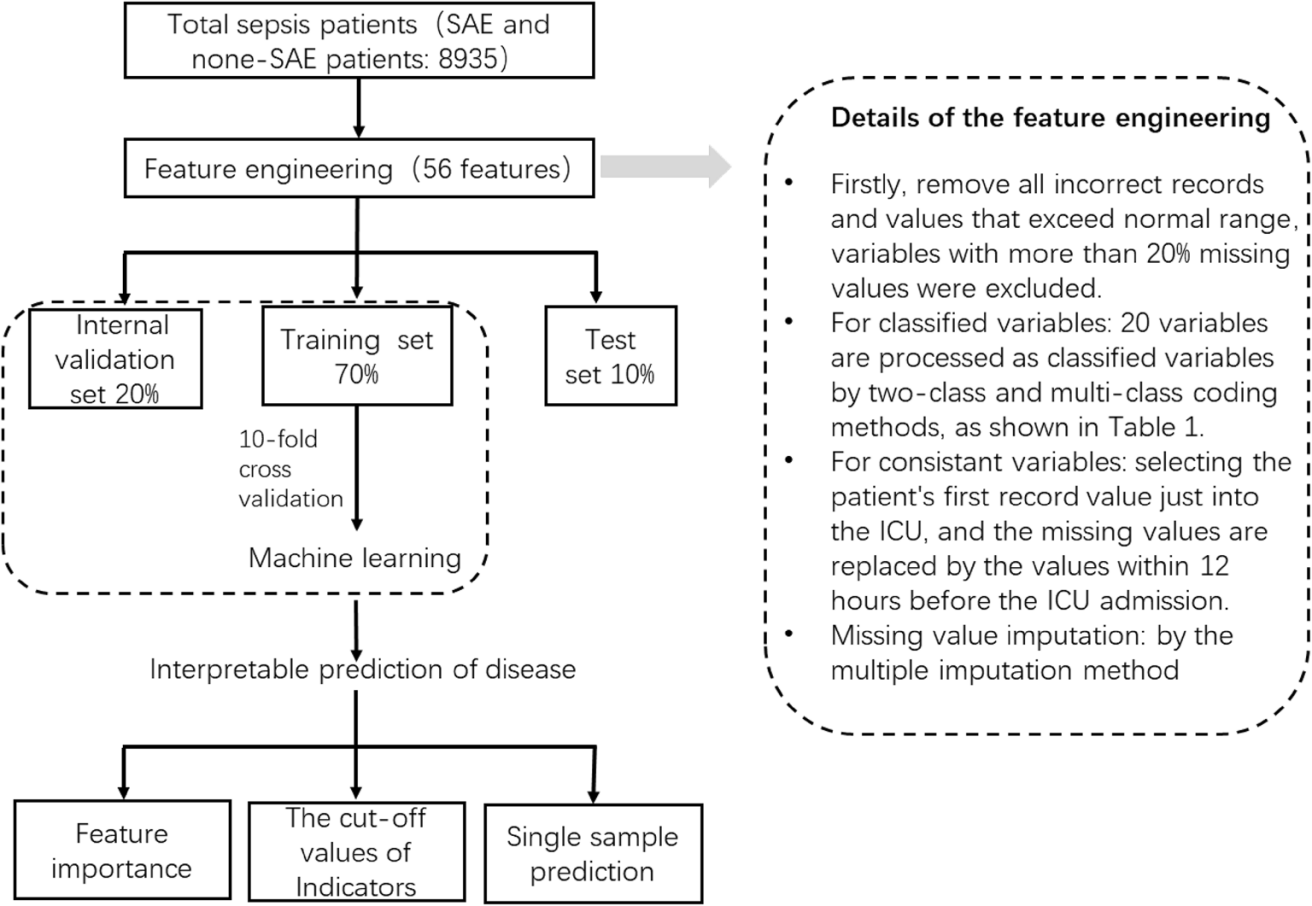
Research flow chart. Details of feature engineering and machine learning prediction processing.

The scheme of the overall experimental design process was shown in Fig. 2. Firstly, according to the data inclusion criteria, the corresponding data would be extracted and cleaned. Then these features were fed into different machine learning classifiers to choose the best model. We randomly split the data of SAE and non-SAE patients by a 7:2:1 ratio for training, internal validation, and testing respectively, and tenfold cross validation was adopted. We randomly set aside a group of 10% data for final testing, tenfold cross validation was just used for the remaining 90% of the data. Six machine learning classifiers were employed to predict the occurrence of SAE, and they are Gradient Boosting Decision Tree (GBDT), Extreme Gradient Boosting Model, Random Forest (RF), Light Gradient Boosting Machine (Light-GBM), Decision Tree (DT), and Support Vector Machines (SVM). The performance of the different classifiers was compared by the area under the Receiver Operating Characteristic Curve (ROC). To identify potentially relevant features for the occurrence of SAE of the study participants and make the model interpretable, the Shapley additive explanation (SHAP)^23^ was utilized to analyze the feature importance and cut-off values, and finally make interpretable predictions for a single sample. The SHAP was based on game theory and can transform the model into a sum effect of all feature attributes to obtain the prediction. Moreover, the effect of each feature on the final prediction can be measured by the SHAP value. The SHAP installation package and the machine learning model packages were imported in a python3.7 environment, and can be referred from the official website: https://shap.readthedocs.io/en/latest/api.html.

### Statistical analysis

Data were presented in the Table1 according to different types and distributions of variables. The completeness of the features we chose was above 80%, and we used multiple interpolation methods to fill in the missing value. The demographic and baseline characteristics of the study population were compared using the Pearson chi-square test for categorical variables and Student’s *t*-test for continuous variables. Normality tests were performed using the Shapiro–Wilk test. Normally distributed continuous variables, non-normally distributed continuous variables, and categorical variables were expressed as mean ± standard deviation, quartiles, and count or percentage, respectively; differences were detected using the two-sample independent *t*-test, rank sum test, and chi-square test, respectively. SPSS software for Windows (version 25.0, SPSS Inc., Chicago, IL, USA) was used for the statistical analyses. An alpha level of 0.05 was set for statistical significance.

### Model performance evaluation method

We used AUC-ROC, AUC-PR, AUC, sensitivity, specificity and F1, which were commonly used in machine learning to evaluate and compare the model performance. SHAP was used to explain the model prediction results. To further evaluated the interpretability of the model, we invited six neurosurgeons and ICU physicians to score the prediction results of our model. The physicians scored the cut-off points of the significant indicators from Fig. 4c, and then offered values from their own medical perceptions. By comparing the results of model interpretation with the evaluation of physicians, we can make an objective clinical evaluation of the interpretable model.

### Ethical approval

The authors are accountable for all aspects of the work in ensuring that questions related to the accuracy or integrity of any part of the work are appropriately investigated and resolved. The data used are from publicly available datasets.

The Institutional Review Board at the Beth Israel Deaconess Medical Center waived the informed consent to the study because the project did not impact clinical care and all protected health information was deidentified. The study conformed to the provisions of the Declaration of Helsinki (as revised in 2013). The study protocol was approved by Beijing Institute of Technology.

## Results

### Statistical analysis of baseline difference between SAE and non-SAE group

A comparative analysis between the SAE group and non-SAE group was performed in terms of demographics, race, first care unit, severe score, comorbidities, physical, laboratory results and microorganisms. The baseline characteristics of the two groups are displayed in Table 1. Analysis of demographic characteristics presented that the SAE group and the non-SAE group had little difference in gender, while the SAE patients were older and mostly occurred in the Asian population, compared to white, black, Hispanic, and Latino. In addition, it revealed that the first care unit category of SAE patients was mainly TSICU and SICU, and the SAE group had a more serious medical score than the non-SAE group. As for comorbidities, SAE patients had a lower incidence of diabetes (26.3 vs. 30.7%). SAE group was also found to be statistically different from the non-SAE group in the vital signs such as respiration, temperature, partial pressure of oxygen, and mean arterial pressure, but not significantly different in the mean values. Regarding to laboratory events, all indicators included in the analysis had statistical differences except phosphorus, platelet count, PCO_2_, pH, BUN, and PT. Statistically significant differences were observed in microbial infection including gram-positive, gram- negative and fungus. Meanwhile, SAE patients had a higher rate of mechanical ventilation and vasopressor use than non-SAE group. Besides, SAE group had approximately twice ICU stay time as the non-SAE group.

### Best cutoff point for risk factors identified by the optimal classification model

**Figure 3:**
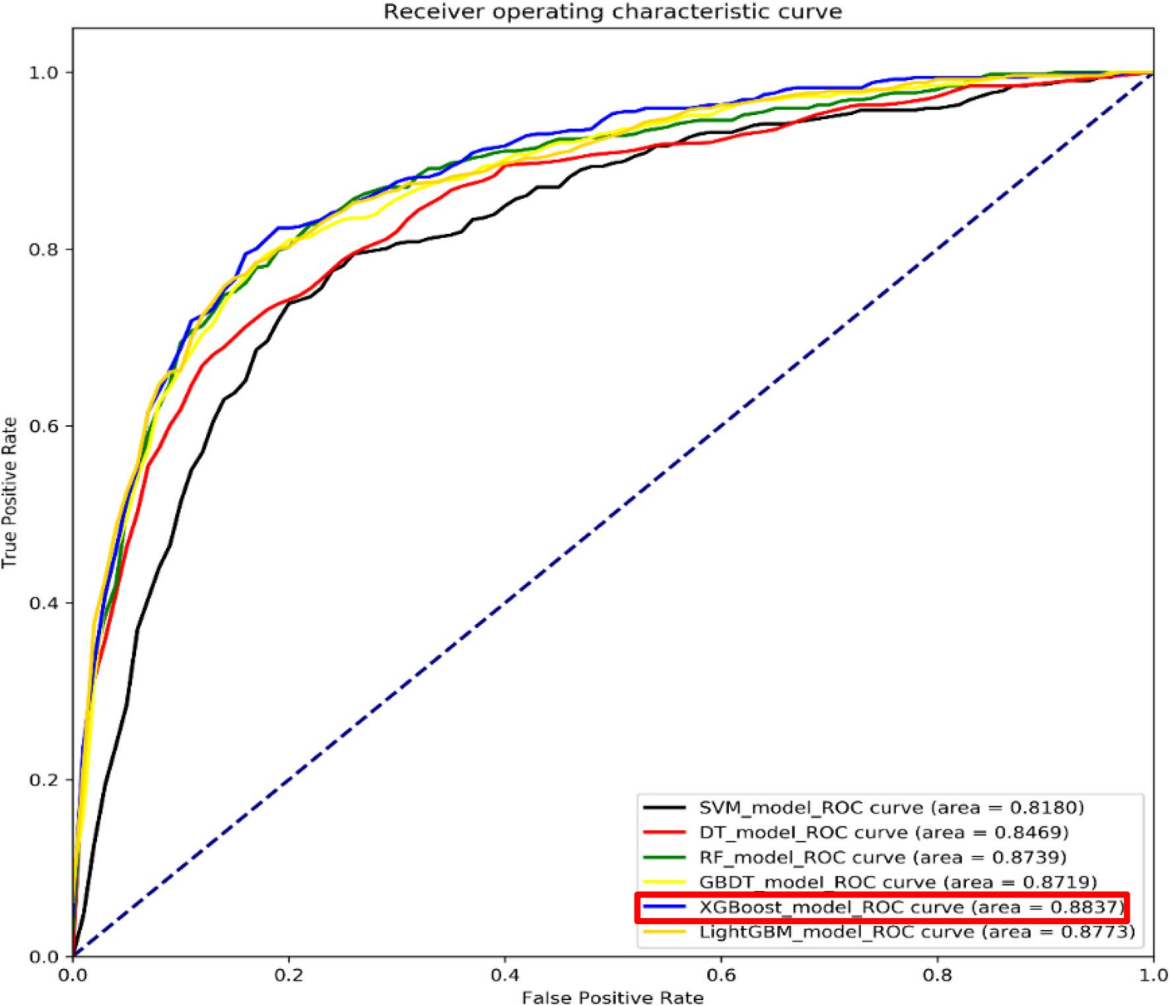
Comparison of prediction performance of six machine learning classification algorithms. The optimal model is XGBoost in the red box. The six algorithms include Gradient Boosting Decision Tree (GBDT), Extreme Gradient Boosting (Xg-boost), Random Forest, Light-GBM, Decision Tree, and Support Vector Machine (SVM). The ROC curve of GBDT is shown by the yellow line, the ROC curve of Xg-boost is shown by the blue line, the ROC curve of Random Forest is shown by the green line, the ROC curve of Light-GBM is shown by the orange line, the ROC curve of Decision Tree is shown by the red line, the ROC curve of SVM is shown by the black line.

**Figure 4:**
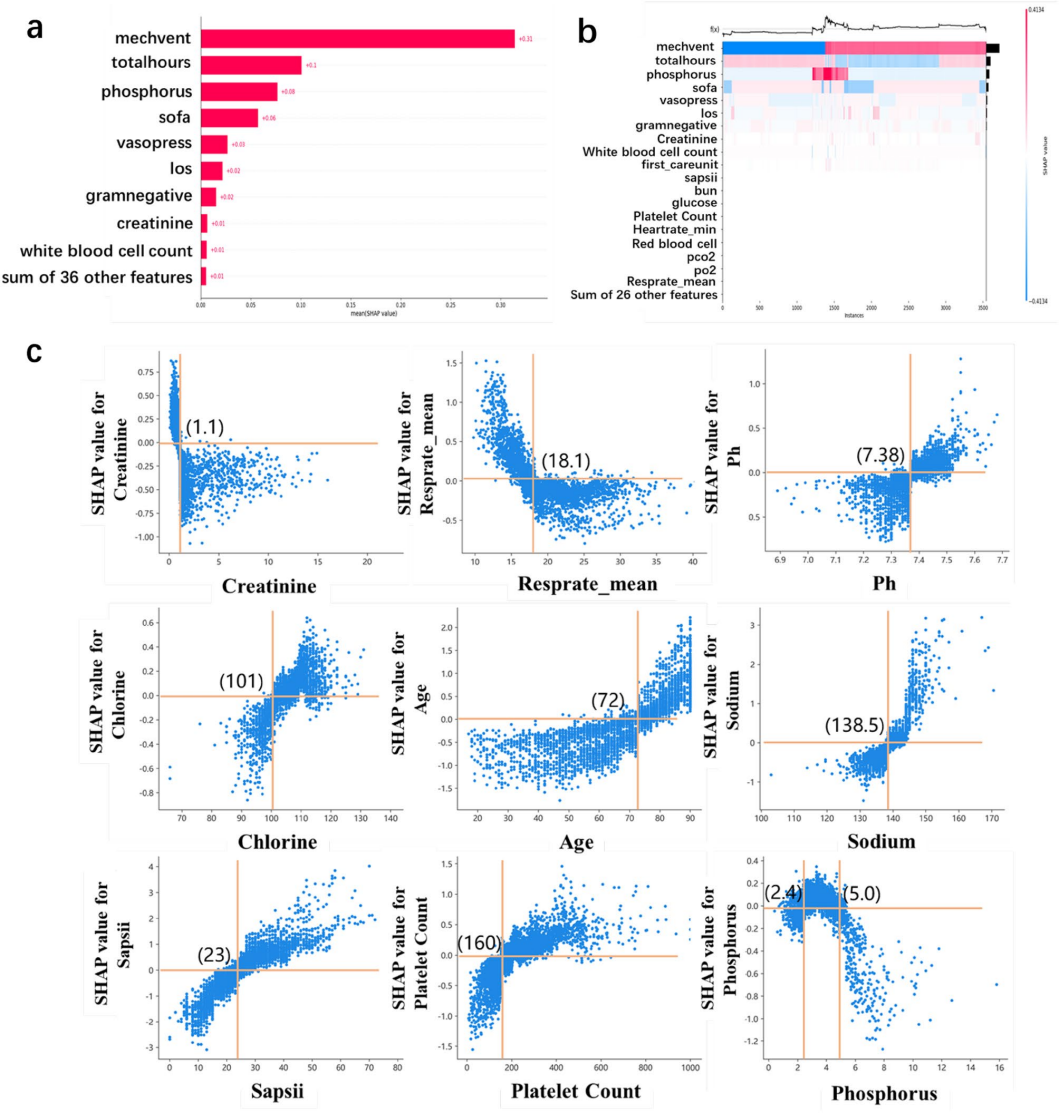
SHAP diagram of the model. (**a**) Feature importance ranking (the global importance of each feature is considered to be the average value of the feature in all given samples). (**b**) Matrix heat map of SHAP value. (**c**) The cut-off points of SAE risk factors obtained by the best classification model.

As shown in Fig. 3, six machine learning algorithm models were implemented to classify the patients into SAE and non-SAE groups. According to the comparison of the model’s performance, eXtreme Gradient Boosting (XGBoost) had the best performance with 0.884 for AUC-ROC, 0.894 for F1-score, 0.615 for AUC-PR, 0.875 for sensitivity, 0.782 for specificity, while SVM was the worst with an AUC-ROC of 0.818. The Table 2 shows the comparing results of the six models and the 95% confidence interval on the training and testing dataset respectively. GBDT, XGBoost, SVM and Random Forest perform better on the training set than on the testing set, while Light-GBM and decision tree perform little differently on the two data sets. No matter on the testing set or the training set, XGBoost 's prediction results are better than other models. The output of the XGBoost model can be interpreted by the SHAP values, which provide a fair measurement for the role of each feature and the effect on the model. Model feature importance ranking was illustrated in Fig. 4a, with the top five being mechanical ventilation, duration of mechanical ventilation, phosphorus, SOFA score, and vasopressor usage. As depicted in Fig. 4b, for the same risk factors, the samples with the same output are stratified and clustered according to the explanatory similarity. The horizontal axis represented each sample and the vertical axis represented the SHAP values. Furthermore, scatter plot based on SHAP values in Fig. 4c indicated the best cut-off points of SAE risk factors, they were creatinine (1.1 mg/dl), mean respiratory rate (18), pH (7.38), age (72), chlorine (101 mmol/L), sodium (138.5 k/u), SAPSII score (23), platelet count (160), and phosphorus (2.4 and 5.0 mg/dL). The blue sample points above 0 on the Y-axis represented a high risk of SAE.

**Table 2:** Performance evaluation of the different machine learning models in training and testing dataset.

Training Dataset					
Models	AUC-ROC (95%CI)	AUC-PR (95%CI)	Sensitivity (95%CI)	Specificity (95%CI)	F1-score (95%CI)
GBDT	0.883 (0.847–0.896)	0.593 (0.567,0.601)	0.874 (0.863–0.880)	0.745 (0.731–0.762)	0.895 (0.879–0.911)
Xg-boost	0.902 (0.883–0.919)	0.632 (0.618,0.644)	0.894 (0.877–0.909)	0.791 (0.775–0.812)	0.901 (0.892–0.915)
Light-GBM	0.879 (0.864–0.887)	0.542 (0.524,0.553)	0.879 (0.861–0.887)	0.789 (0.771–0.795)	0.893 (0.872–0.908)
SVM	0.832 (0.824–0.857)	0.578 (0.569,0.592)	0.891 (0.872–0.909)	0.789 (0.763–0.797)	0.849 (0.832–0.859)
Decision tree	0.849 (0.838–0.868)	0.589 (0.561,0.604)	0.869 (0.853–0.871)	0.723 (0.711–0.749)	0.861 (0.843–0.871)
Random forest	0.886 (0.871–0.890)	0.542 (0.531,0.559)	0.868 (0.841–0.879)	0.781 (0.767–0.798)	0.893 (0.872–0.905)

Testing Dataset					
Models	AUC-ROC (95%CI)	AUC-PR (95%CI)	Sensitivity (95%CI)	Specificity (95%CI)	F1-score (95%CI)
GBDT	0.872 (0.859–0.885)	0.574 (0.548,0.582)	0.866 (0.842–0.873)	0.723 (0.712–0.745)	0.886 (0.873–0.905)
Xg-boost	0.884 (0.865–0.898)	0.615 (0.587,0.623)	0.875 (0.865–0.879)	0.782 (0.764–0.808)	0.894 (0.886–0.912)
Light-GBM	0.877 (0.869–0.888)	0.523 (0.504,0.547)	0.863 (0.850–0.876)	0.774 (0.769–0.781)	0.882 (0.861–0.897)
SVM	0.818 (0.808–0.839)	0.565 (0.538,0.576)	0.871 (0.861–0.889)	0.782 (0.748–0.796)	0.827 (0.801–0.844)
Decision tree	0.847 (0.839–0.855)	0.582 (0.563,0.594)	0.860 (0.852–0.871)	0.719 (0.706–0.743)	0.858 (0.842–0.861)
Random forest	0.874 (0.868–0.881)	0.533 (0.518,0.551)	0.847 (0.830–0.858)	0.778 (0.761–0.794)	0.884 (0.860–0.893)

CI: Confidence interval; AUC: area under the receiver operating characteristic curve.

### Personalized prediction and model output interpretation

**Figure 5:**
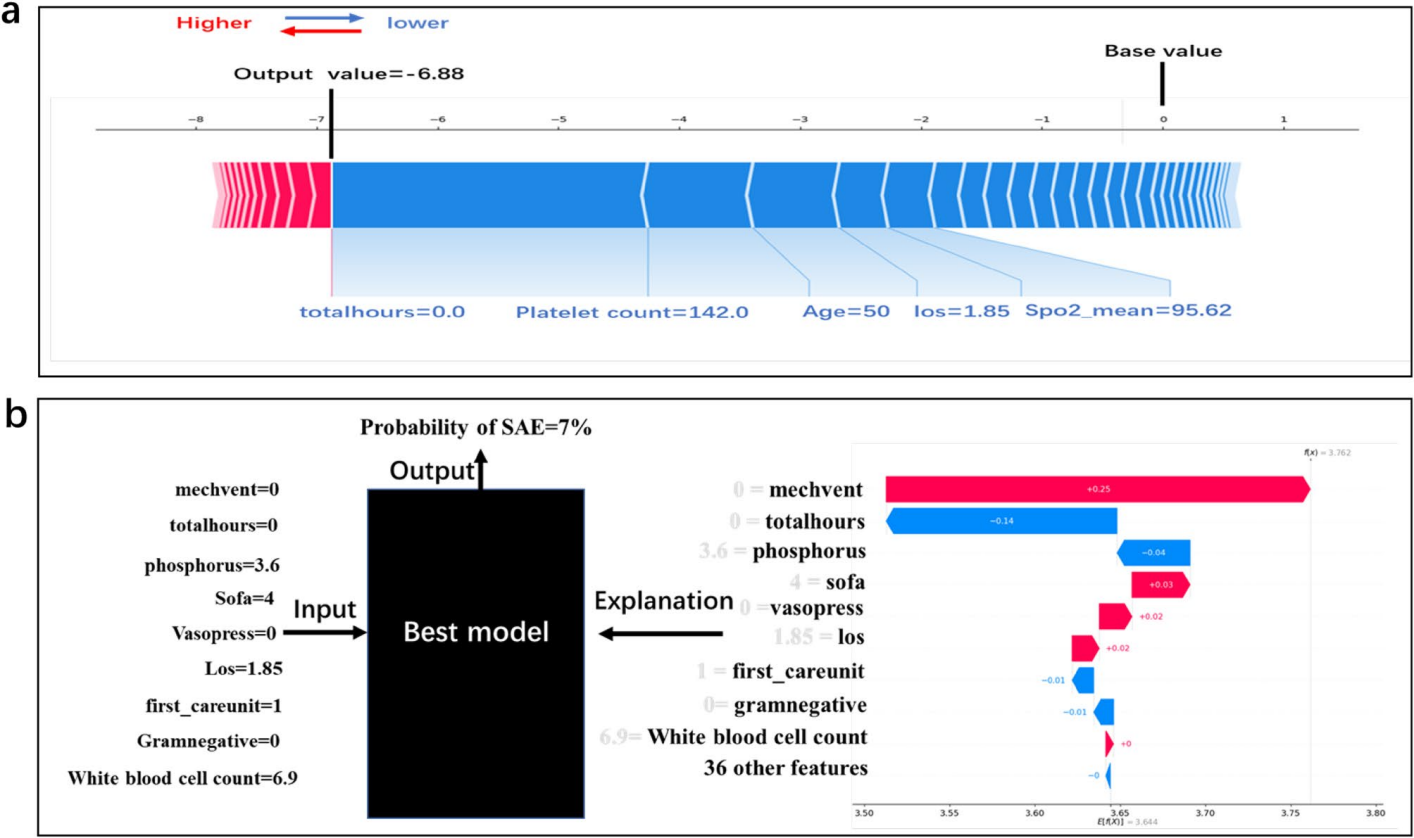
Prediction and interpretation of result for a single sample. (**a**) Characteristic SHAP value influence diagram of a single sample. (**b**) Model prediction diagram and explanation for individual.

We applied the SHAP method to explain how the XGBoost model predicted a single particular instance, Fig. 5a displays a specific example, where the red and blue features represent risk factors and protective factors, respectively. In terms of shape values, longer bars meant more importance. As observed in Fig. 5a, this example is a low-risk instance because the blue features push the risk value of the instance below the base value. Figure 5b shows the probability of SAE after a patient’s indicators are inputted into the model and the impact of each indicator on the model’s results. The random input of a patient’s indicators shows that the probability of this patient suffering from SAE is 7%, and the interpretation of the model is presented on the right, indicating that the absence of mechanical ventilation is the main factor for the model output of low risk of SAE.

### Assessment of the interpretable model prediction results by professional physicians

**Figure 6:**
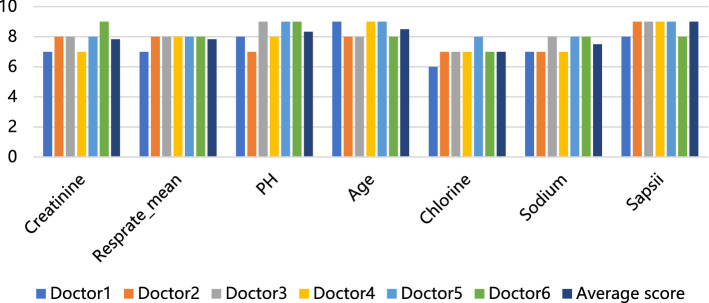
Doctor’s evaluation of the results of model interpretation.

Since the disease prediction model is intended for physicians or patients, we invited six neurosurgeons and ICU physicians to score the prediction results of our model, so as to further quantify the rationality of the interpretability of the model. The physicians scored the cut-off points of the significant indicators (with a maximum score of 10 for each indicator) and then offered values from their own medical perceptions (refer to supplementary Table 1). As shown in Fig. 6, the evaluated scores for the six indicators from six physicians range from 7 to 9, which is generally consistent with physicians' cognition. Therefore, the prediction results of our model are in good agreement with the scores of physicians.

## Discussion

In most previous studies, the occurrence of sepsis and death from sepsis have been predicted, but still there was few predictive studies on the occurrence of sepsis-associated encephalopathy by interpretable machine learning method. In our study, an interpretable machine learning model was established to predict the occurrence of SAE in septic patients within 24 h after entering ICU.

The pathogenesis of SAE is complex, and there is no more systematic diagnostic method in clinical practice. SOFA and LODS have been proved useful tools for predicting short-term mortality in patients with sepsis, but the question that whether they are applicable to SAE is unclear^24,25^. In a study by Yang et al., in the column line graph prediction of the risk of death in SAE based on the MIMIC-III database model, the area under the working characteristic curve was 0.763 and 0.753 for subjects in the training and validation groups, respectively. Zhao et al.^26^ investigated the diagnostic and prognostic value of serum tau levels in predicting the development of SAE in patients with sepsis, and the AUC was 0.770. At the same time, the study pointed out that combining the level of serum tau protein with SOFA score can lead to further improvement in performance, with the AUC of 0.798. Therefore, our developed predictive model based on 56 clinical characteristics such as demographics, race, severity score, comorbid diseases, and vital signs of sepsis patients has a excellent predictive effect on the occurrence of SAE, the AUC metrics of all six models were all above 0.8, and the XGBoost model had the optimal performance (AUC was 0.884).

In medical research, inaccurate prediction results of machine learning models often bring more serious consequences, so we should pay much more attention to the judgment basis of model prediction and judge whether it can output reliable prediction results. In this study, we used the SHAP value to perform an interpretable analysis of the optimal model XGBoost to meet the needs of clinicians to understand the model output as well as personalized prediction and improve the model credibility. Although the feature importance and the cutoff values were very different for six different machine learning after the shap interpretation, the interpretation results of shap value were little different for the tenfold cross validation of the same model, so we randomly chose results of one time cross validation to present. After the XGBoost model was ranked by feature importance, the top five were mechanical ventilation or not, duration of mechanical ventilation, phosphorus, SOFA score, and vasopressor use. In addition, we plotted scatter plots based on SHAP values, the influence of some features (such as pH, age, and SAPSII scores) on the final output of the model resulted in the higher the value, and the more likely the patient was predicted to be SAE. While some features such as respiratory rate (Resprate_mean), their influence on the model was that the relatively lower the value, the higher the likelihood the patient was predicted to be SAE. Also, the cut-off point is given to provide the critical values of important indicators. Therefore, the SHAP graph-based interpretation method visualizes the contribution of individual features to the model output and is also important in the personalized prediction of clinical decisions. Since the physicians or patients are the direct application target of disease prediction models, the results of our prediction model were evaluated by physicians to make our model more convincing for them. Because physicians judge diseases from a combination of aspects, and the importance indicators calculated by machine learning are different from those considered by physicians, physicians only give the evaluation of six indicators out of their own perception. The final scores from the physicians are relatively high, indicating that our prediction model can provide a reasonable interpretation and can be reliably used for predicting new patients in new samples.

Since the assessment of SAE in clinical practice using traditional diagnostic methods is time-consuming and complex, the machine learning model we developed aims to assist clinicians in diagnosing and treating SAE in a timely manner while reducing the burden on medical resources. On the other hand, the interpretability of the machine learning model can help physicians and patients clearly understand the model decision process, take the prediction and associated importance features for an individual case.

This study also had some limitations. Firstly, this study was conducted based on the MIMIC-IV database, which is relatively homogeneous. And we only performed internal validation through this database, and some external databases should be considered in the future to further validate the robustness and performance of the model. In addition, only the importance of a single feature is shown in the interpretability analysis of the model, while it is also important to understand the interactions between features in the actual prediction process.

## Conclusion

In this retrospective study, we proposed an interpretable machine learning model for predicting the occurrence of SAE within 24 h after admission to ICU. All features important to the model are derived from clinical variables routinely collected. The interpreting model can help physicians and patients to determine the occurrence of SAE more visually.

## Supplementary Information

Supplementary Information.

## Data Availability

The MIMIC IV database (version 0.4) is publically available from https://physionet.org/content/mimiciv/0.4/. The raw data were extracted using structure query language (SQL) with navicat and further processed with python 3.7.

## Abbreviations

SAE: Sepsis-associated encephalopathy
ICU: Intensive Care Unit
SHAP: Shapley Additive explanation
XGBoost: EXtreme Gradient Boosting
SOFA score: Sequential Organ Failure Assessment
PH: Potential of hydrogen
SAPSII score: Simplified Acute Physiology Score
