# Supplementary material for: Prediction and risk assessment of sepsis-associated encephalopathy in ICU based on interpretable machine learning
Source: Sci Rep. 2022 Dec 31;12:22621. doi: 10.1038/s41598-022-27134-6 (PMC9805434; doi:10.1038/s41598-022-27134-6)
Supplement: Supplementary file 1 — Supplementary Information. [file 41598_2022_27134_MOESM1_ESM.docx]

**Supplementary table 1 Doctor’s evaluation of the results of model interpretation**

| **Evaluation Specialist** | **Creatinine** | **Resprate_mean** | **PH** | **Age** | **Chlorine** | **Sodium** | **Sapsii** | **Average score** |
| --- | --- | --- | --- | --- | --- | --- | --- | --- |
| Doctor1 | 7 | 7 | 8 | 9 | 6 | 7 | 8 | 7.43 |
| Doctor2 | 8 | 8 | 7 | 8 | 7 | 7 | 9 | 7.71 |
| Doctor3 | 8 | 8 | 9 | 8 | 7 | 8 | 9 | 8.14 |
| Doctor4 | 7 | 8 | 8 | 9 | 7 | 7 | 9 | 7.86 |
| Doctor5 | 8 | 8 | 9 | 9 | 8 | 8 | 9 | 8.43 |
| Doctor6 | 9 | 8 | 9 | 8 | 7 | 8 | 8 | 8.14 |
| Average score | 7.83 | 7.83 | 8.33 | 8.50 | 7.00 | 7.50 | 8.67 | 7.95 |

Full score for each evaluation is 10 points
